# Conditional Adversarial Domain Adaptation Neural Network for Motor Imagery EEG Decoding

**DOI:** 10.3390/e22010096

**Published:** 2020-01-13

**Authors:** Xingliang Tang, Xianrui Zhang

**Affiliations:** 1 School of Information Science and Engineering, LanZhou University, Lanzhou 730000, China; 2 Sichuan Jiuzhou Electric Group Co Ltd, Mianyang 621000, China; 3 Department of Automation Sciences, Beihang University, Beijing 100191, China

**Keywords:** electroencephalogram (EEG), motor imagery (MI), domain adaptation, signal classification, convolutional neural network

## Abstract

Decoding motor imagery (MI) electroencephalogram (EEG) signals for brain-computer interfaces (BCIs) is a challenging task because of the severe non-stationarity of perceptual decision processes. Recently, deep learning techniques have had great success in EEG decoding because of their prominent ability to learn features from raw EEG signals automatically. However, the challenge that the deep learning method faces is that the shortage of labeled EEG signals and EEGs sampled from other subjects cannot be used directly to train a convolutional neural network (ConvNet) for a target subject. To solve this problem, in this paper, we present a novel conditional domain adaptation neural network (CDAN) framework for MI EEG signal decoding. Specifically, in the CDAN, a densely connected ConvNet is firstly applied to obtain high-level discriminative features from raw EEG time series. Then, a novel conditional domain discriminator is introduced to work as an adversarial with the label classifier to learn commonly shared intra-subjects EEG features. As a result, the CDAN model trained with sufficient EEG signals from other subjects can be used to classify the signals from the target subject efficiently. Competitive experimental results on a public EEG dataset (High Gamma Dataset) against the state-of-the-art methods demonstrate the efficacy of the proposed framework in recognizing MI EEG signals, indicating its effectiveness in automatic perceptual decision decoding.

## 1. Introduction

Electroencephalogram (EEG) signal decoding is an important part in a brain–computer interface (BCI) system, which establishes a direct communication pathway between the human brain and external devices by translating neuronal activities into a series of output commands to accomplish the user’s intentions [1]. Thereby, it has a wide range of applications from clinic to industry for both patients and normal people [2], such as controlling a wheelchair or prosthesis to improve the life quality of the disabled [3], affecting neural plasticity to facilitate stroke rehabilitation [4], and handling computer games for the entertainment of healthy users [5]. Despite impressive advancements in recent years, EEG-BCI technology is still not able to decode complicated human mental activities’ effectiveness, due to the high complexity of cognitive processing procedures in the brain and a low signal-to-noise ratio in EEG signals. Therefore, it is imperative to develop an effective EEG decoding scheme for enhancing the usability of BCI systems.

In recent years, many machine learning methods have been proposed to decode EEG signals. These methods mainly include two stages. Firstly, discriminative features, such as entropy feature sets [6], the frequency band power [7], and the filter band common spatial pattern (FBCSP) [8,9] are extracted from each EEG trial. Then, these informative features are fed into classifiers, including a support vector machine (SVM) and random forest, to generate the final recognition result [10,11]. However, these two-stage methods heavily rely on handcrafted features based on a fixed frequency band, which cannot be determined manually for all cases and, consequently, result in a suboptimal classification performance [12]. Thus, some studies try to investigate the capacity of deep learning methods in motor imagery EEG decoding because of its excellent performance in image processing [13], speech recognition [14], and so forth. Stober et al. combined stacked denoising autoencoders and a convolutional neural network (ConvNet) to classify decomposed spectral features, which are produced by a frequency band analysis of EEG signals [15]. Tabar et al. proposed a novel deep network which combines ConvNet and stacked autoencoders to extract time-frequency features and classify motor imagery EEG signals [16]. The classification results of this novel deep network outperformed the traditional FBCSP algorithm [17]. Recently, ConvNet architecture was also employed to detect multi-channel P300 waves [18] and rapid serial visual presentation tasks [19]. More recently, in [12], Schirrmeister et al. introduced a novel Deep ConvNet, a Shallow ConvNet, and a Residual ConvNet to recognize four-class motor imagery EEG signals. Especially, the proposed Deep ConvNet gained state-of-the-art results (with an accuracy of 92.5% on the four-class motor imagery tasks) on the public High Gamma Dataset.

However, considering the inconvenience of acquiring labeled EEG data, an important methodological problem still hinders the application of ConvNet in EEG decoding, namely, the requirement of sufficient labeled EEG data for training [20]. Therefore, it is worth proposing a deep learning architecture that could be trained using the abundant EEGs from other subjects applied to recognize EEG signals from the target subject directly [21]. However, considering the substantial variabilities between inter-subject EEG data, the mismatch of the extracted EEG feature distributions between the source and the target subjects may consequently lead to poor EEG decoding performance [22]. In this paper, we assume that the electrode placements have little effect on the variability of the EEG signals when collecting EEG signals from the same subject in one experiment. We mainly focus on the variety between different subjects which influence the model’s performance.

Recently, in order to mitigate the discrepancy of different feature distributions, domain adaptation techniques have been applied by using sufficient EEG signals from subjects in source domains to boost the model’s performance on the target subject [23]. Typically, EEG trials with class labels from other subjects are defined as the source domain, while EEG trials without labels from the target subject are called the target domain. Traditional domain adaptation techniques, such as transfer component analysis [24], mainly focus on minimizing the discrepancy of the feature distribution on the basis of the hand-engineered EEG features using a distance metric and, finally, the shared common feature representation between different subjects are extracted. More recently, a covariate shift adaptation method was introduced to analyze a nonstationary EEG time series from different sessions of a public BCI competition dataset [25]. However, the aforementioned methods still primarily rely on the quality of handcrafted features from EEG signals. On the contrary, Li et al. proposed a new deep learning model for EEG emotion recognition between different subjects: the bi-hemisphere domain adaptation network (DANN), which extracts common features between different subjects [26]. It introduces a domain discriminator and a feature extractor to mitigate the variability of the learned feature representation and extracts commonly shared EEG features by minimizing the distribution discrepancy between the EEG signals from the source and target subjects. Finally, the label information of the signals from the target subject can be predicted much more accurately on the basis of the classifier trained by EEG signals from source subjects.

Despite its general efficacy for EEG signal recognition, the adversarial domain adaptation method for EEG patterns is still constrained by two main challenges, namely, the decrease of mismatch in the domain discrepancy and the learning of discriminative and common features across different subjects [27]. If there are complex multimodal structures in the data distributions of EEG signals, these adversarial adaptation techniques cannot capture the potential features because of the equilibrium challenge of adversarial learning [28]. Fortunately, recent advances in the conditional generative adversarial network (CGAN) illustrate that conditioning the generator and discriminator on discriminative information can make distributions of real and fake samples much more similar [29]. Motivated by this, we further extend a variation of CGAN to exploit the EEG features that are more transferable and discriminative and boost the EEGs decoding performance.

In this paper, to tackle the challenges mentioned above, we introduce the conditional domain adversarial neural network (CDAN) for EEG decoding for the first time. Concretely, we firstly apply a dense connect ConvNet for extracting EEG features. Next, the features are fed into a conditional domain discriminator and label classifier, respectively. Additionally, on the basis of classifier predictions, we give priorities to easy-to-transfer examples by allocating higher weights during training, which helps to minimize the distribution mismatch of the extracted features from the source and target EEG datasets. We evaluate the decoding performance of our proposed CDAN method on one public motor imagery EEG dataset collected from 14 subjects. Experimental results demonstrate that our proposed EEG decoding architecture obtains significantly higher classification accuracy than other state-of-the-art deep learning methods. The main contributions of this paper are as follows:

(1) Instead of the classical adversarial adaptation methods, we introduce a novel conditional adversarial adaptation network to classify the cross-subject EEG signals.
(2) A special dense connected ConvNet is introduced to learn discriminative abstract features from raw EEG signals.
(3) For each EEG trial, the uncertainty in loss function is quantified by the entropy criterion calculated by classifier predictions.

As a result, a consistent improvement with different subjects is achieved by our proposed scheme for EEG signal decoding.

The structure of this article is as follows. In Section 2, we introduce the components of the proposed CDAN in detail. In Section 3, we present the experimental results on one public EEG dataset (HGD dataset) and explain the efficiency of the CDAN for EEG decoding. Finally, we summarize and conclude the proposed method in this paper.

## 2. Methods

In this section, firstly, we present the definitions and notations that we use in this paper. Then, we introduce the ConvNet architecture which is taken for the feature extraction. Next, the conditional adversarial domain adaptation method is proposed for learning discriminative subject independent features from raw EEG signals. Finally, we summarize the proposed decoding framework for motor imagery EEG decoding.

### 2.1. Definitions and Notations

Before addressing the details of the proposed decoding architecture, we firstly describe the notations and corresponding definitions that we will use in later sections.

The data collected from one subject is defined as $\left\{\left(X_{i},y_{i}\right),i=1,2,\;\ldots \;n\right\}$, where n represents the number of motor imagery EEG trials. $X_{i}$ ∈ c×t denotes an EEG trial with *c* channels and *t* sampling indexes and $y_{i}$ is the corresponding label. In this paper we evaluate our method on the HGD dataset, which we will describe detailly in Section 3.1. The HGD has four-class motor imagery tasks: left-hand, right-hand, feet, and rest. We define that for $\forall y_{i}:\;y_{i}\in L,L=\{l_{1}=“\mathrm{left}\;\mathrm{hand}”,l_{2}=“\mathrm{right}\;\mathrm{hand}”,l_{3}=“\mathrm{feet}”,\;l_{4}=“\mathrm{rest}”\}$.

In this study, the signals from all the subjects are used as the input of the CDAN. More specifically, when training the model for the $j^{th}$ subject, the EEG trials of other subjects are defined as the source domain, and trials from the $j^{th}$ subject are defined as the target domain. In the source domain, there are $n_{s}$ labeled trials with class labels, which are denoted as $D_{s}={\left\{\left(X_{i}^{s},y_{i}^{s}\right)\right\}}_{i=1}^{n_{s}}$. Similarly, EEG trials collected from $j^{th}$ subject are defined as the target domain $D_{t}={\left\{\left(X_{j}^{t}\right)\right\}}_{j=1}^{n_{t}}$. We train the CDAN model using all the data and labels from the source domain, while in the target domain, the label information is not needed. As for the components in the CDAN, we denote function G(·) as the feature extractor,D(·) as the conditional discriminator, and C(·) as classifier.

### 2.2. Conditional Domain Adaptation Network

**Figure 1:**
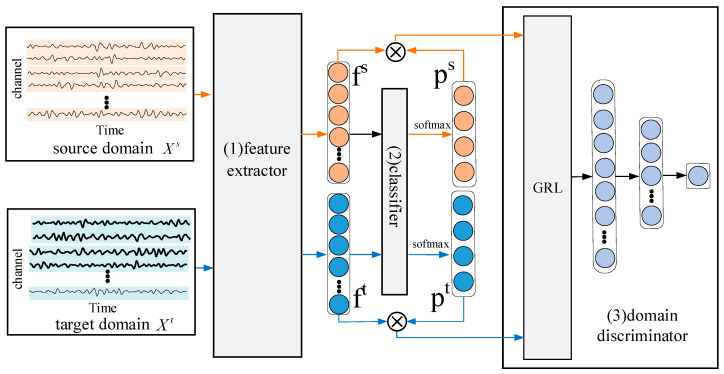
The architecture of the CDAN model.

In order to alleviate the possible differences of feature distributions between EEG signals in the source and target domains, we propose a CDAN model which extracts features by a Dense ConvNet and applies an adversarial operation between source and target domains [30]. As shown in Figure 1, the proposed CDAN model includes three basic subnetworks: (1) feature extractor; (2) classifier, and (3) conditional discriminator. Specifically, the CDAN model employs a three-step strategy during training. Firstly, a batch of EEG signal matrixes consisting of $X^{s}$ and $X^{t}$ are transformed into the high-level discriminative features $f^{s}$ and $f^{t}$ by the feature extractor. Then, these features are typically fed into the classifier to obtain the conditional probabilities vectors $p^{s}$ and $p^{t}$ with the softmax function [7]. Finally, the conditional features are calculated on the basis of the features and probabilities generated above, and the conditional discriminator is introduced to distinguish the EEG data from the source domain or target domain, in which the domain labels of the target dataset are set to 0 and, correspondingly, the domain label of the source is set to 1. The purpose of using a gradient reversal layer (GRL) in the discriminator is to maximize the loss of discriminators by multiplying the gradient with a negative value to reverse it in the backpropagation stage, so that the feature extractor can adversarially learn common EEG features between $X^{s}$ and $X^{t}$ by minimizing the data distribution discrepancy. Therefore, the training of the CDAN is a two-player game [31]: firstly, the conditional discriminator is trained to distinguish the EEG data from the source domain or target domain. Then, the second player, which is the feature extractor, is employed simultaneously to confuse the conditional discriminator and stop it from recognizing the domain of EEG signals correctly. As a consequence, the feature distribution of the source domain can be aligned with that of the target domain accurately, so that the trained model can learn common features from different domains and be used to recognize EEG signals from both the target domain and the source domain. In the following, we will introduce the feature extractor, classifier, and conditional discriminator detailly.

#### 2.2.1. Feature Extractor

**Figure 2:**
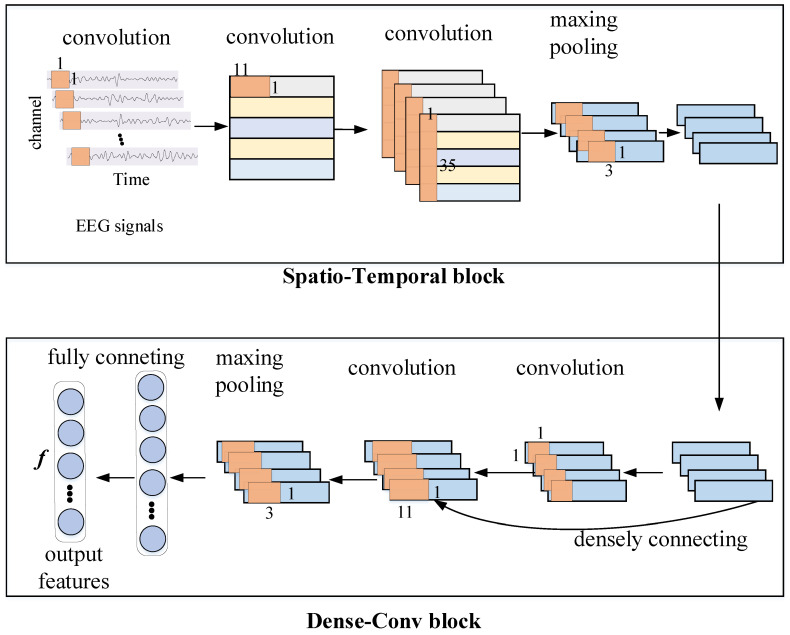
Illustration of the Dense ConvNet architecture, where the blue cuboids represent feature maps, and the browns are the kernels of convolution and pooling.

In this section, we employ the architecture of Dense ConvNet to implement the feature extractor, which includes the Spatio-Temporal block and the Dense-Conv block. A diagram of the Dense ConvNet is shown in Figure 2. Firstly, the multi-channel EEG signals are fed into the model as input. Then, the Spatio-Temporal block is applied to learn temporal and spatial representations from the EEG signals. Finally, the Dense-Conv block is introduced to learn discriminative features by a short-cut path in the architecture.

As is illustrated in Figure 2, the Spatio-Temporal block mainly includes the channel-projection layer, the temporal convolutional layer, and the spatial convolutional layer. Assuming that we get a trial of MI EEGs with *c* channels and *t* time points, it is then firstly reshaped as a matrix with a size of c × 1 × t as input before being fed into the channel-projection layer. We transpose it to c × 1 × t and put the channel at the first dimension in the EEG matrix, as this would be convenient for a later 1 × 1 convolution operation. The first 1 × 1 convolution is applied to change the dimensionality of the signals, meanwhile the spatial dependency in the EEG signals is extracted, so that the 1 × 1 convolution operation can be used to learn the spatial dependency between each EEG electrode and map the number of channels from *c* to 35. The design of this layer is motivated by the operation of FBCSP to extract the spatial features from raw EEG signals [17]. Furthermore, the computing efficiency of the 1 × 1 convolution [32] in the channel dimension of the EEG signals enables the channel-projection layer to handle input signals with a number of channels. Meanwhile, the convolution operation with a size of 1 × 1 ensures that the output feature maps with a size that is consistent with the input EEG representations. Then, the temporal convolution and spatial convolution are employed successively. We set the size of the convolutional kernel in the temporal layer to 25 × 1 and in the spatial layer to 1 × 35 with the stride of 1. Note, that the kernel size of the convolution filter in the proposed CDAN model is set empirically according to the architecture of the deep ConvNet in [12], which has proven that such a size of kernel is proper for the feature extraction of a EEG time series. In addition, the operations of dropout and normalization are inserted after each convolution layer, and the exponential linear unit (ELU) [33] is used as the activation function after the spatial convolutional layer for further robustness and nonlinearity activations. Finally, we employ the maxing pooling operation to generate a group of coarser 1-D intermediate representations.

On the basis of the spatial and temporal dependencies extracted above, the Dense-Conv block is employed to learn high-level and discriminative temporal representation, as shown in Figure 2. More specifically, this block includes one 1 × 1 convolutional layer, one 11 × 1 convolutional layer, and one 3 × 1 maxing pooling layer successively. The densely connected operation [30] is also introduced to stack the input and output feature maps together, as it can provide a short-cut path for the propagation of gradients in the networks in the backpropagation procedure and alleviate the problem of the vanishing gradient during training [34]. The maxing pooling layer follows to reduce the lengths of the output feature maps which are flattened to a 1-D vector and fed into a fully connected layer. As a result, the most discriminative features, which are denoted as *f*, are extracted from the raw EEG time series. Note, that *f* represents both source domain features $f^{s}$ and target domain features $f^{t}$, here.

#### 2.2.2. Classifier and Conditional Discriminator

The aim of the classifier in Figure 1 is to distinguish the high-level features in EEG signals across the source and target subjects and assign labels to each EEG trial correctly according to the output of the softmax function. The classifier is a two-layer, fully connected network with 256 input units and 4 output units. As is shown in Figure 1, it takes feature *f* as input and its output is fed into the softmax function to obtain the predicted probability *p* for each class of EEG signals.

Since only the EEG data from the source domain have labels, the loss of classifier is calculated based on probability $p_{i}^{s}$ and label $y_{i}^{s}$ from the source domain, which is defined as:

(1) $$L_{c}\left(\theta_{f},\theta_{c}\right)=E_{(x_{i}^{s},y_{i}^{s})∼D_{s}}L(p_{i}^{s};y_{i}^{s}),$$

where $\theta_{f}$ and $\theta_{c}$ represent the learned parameters in the feature extractor and classifier, respectively, $p_{i}^{s}$ is the conditional probability vector that softmax function generates, $y_{i}^{s}$ is the corresponding label, and L(⋅) is the cross-entropy loss function.

As is illustrated in Figure 1, the target of the conditional discriminator is to distinguish EEG trials in the source domain from the target domain. With the Sigmoid operation inserted as the activation function, it is a fully connected network which takes the combined conditional features as input and has one output unit. The design of the conditional discriminator is inspired by recent research about the conditional generative adversarial network (CGAN) [29]. The CGAN demonstrates that conditioning the discriminator of the framework on relevant information can match different distributions better. Motivated by this, in [28], it is observed that the classifier softmax output *p* contains discriminative information potentially, and it can be used as the condition when adapting feature representation *f* simultaneously.

As for the input of the conditional discriminator, namely the combined conditional features, one widely applied conditioning strategy is concatenating the feature representation *f* and classifier prediction *p*. However, this method cannot fully exploit the multimodal information contained in the classifier prediction and get multiplicative interactions with feature representation, as *f* and *p* are independent of each other. Instead of this simple concatenating operation, in this paper, the discriminator *D* is conditioned by the multilinear map as:

(2) h= f⊗p,

where ⊗ refers to the operation of multilinear map, *h* is the conditional features which combines the probability *p* and feature representation *f* together. For example, considering the probability *p* = $\left[p_{1},p_{2},p_{3}\cdots p_{d_{p}}\right]$ and feature representation *f* = $\left[f_{1},f_{2},f_{3}\cdots f_{d_{f}}\right]$, respectively. The result *h* of the multilinear map is expressed as:

(3) $$\left[\begin{array}{cccc} f_{1}\cdot p_{1} & f_{1}\cdot p_{2} & \cdots & f_{1}\cdot p_{d_{p}}\\ f_{2}\cdot p_{1} & f_{2}\cdot p_{2} & \cdots & f_{2}\cdot p_{d_{p}}\\ \vdots & \vdots & \ddots & \vdots \\ f_{d_{f}}\cdot p_{1} & f_{d_{f}}\cdot p_{2} & \cdots & f_{d_{f}}\cdot p_{d_{p}} \end{array}\right].$$

The joint variable *h* is used as the input feature of the conditional discriminator and enables it to tackle the challenges of the adversarial domain adaptation, potentially, by modeling the joint distributions of *f* and *p*. The loss function of the discriminator is presented as:

(4) $$L_{d}(\theta_{f},\theta_{d})=-E_{x_{i}^{s}∼D_{s}}\log[D(h_{i}^{s})]-E_{x_{j}^{t}∼D_{t}}\log[1-D(h_{j}^{t})],$$

where $h_{i}^{s}=f_{i}^{s}⊗p_{i}^{s}$ and $h_{j}^{t}=f_{j}^{t}⊗p_{j}^{t}$ are the joint variables of EEG trials from source domain and target domain respectively, $D(h_{i}^{s})$ and $D(h_{j}^{t})$ represent the output of discriminator corresponding to $h_{i}^{s}$ and $h_{j}^{t}$ as input, respectively. For EEG samples from the source domain and target domain, their losses are calculated by $-\log[D(h_{i}^{s})]$ and $-\log[1-D(h_{j}^{t})]$ respectively. The final discriminator loss on the whole dataset is gained by averaging the values from all EEG trials.

However, the same importance for each example in Equation (4) may lead to the conditional adversarial adaptation procedure suffering from the EEG trials with extremely uncertain prediction results. Therefore, we define the entropy criterion as a metric to quantify the uncertainty of classifier predictions. Assuming that we have the probability vector *p* that the softmax function generates, the uncertainty of the predicted probability is formulated as:

(5) $$H(p)=-\sum_{c=1}^{C}p_{c}\log p_{c},$$

where *C* is the number of classes and $p_{c}$ is the $c^{th}$ element in the probability vector *p*. On the basis of the entropy criterion, we reweight each sample by:

(6) $$w(H(p))=1+e^{-H(p)}$$

As a result, the easy-to-transfer examples with lower uncertainty H(p) would have higher weights w, so the loss function in (3) can be modified in the new entropy-based loss function as:

(7) $$L_{d}(\theta_{f},\theta_{d})=-E_{x_{i}^{s}∼D_{s}}w(H(p_{i}^{s}))\log[D(h_{i}^{s})]-E_{x_{j}^{t}∼D_{t}}w(H(p_{j}^{t}))\log[1-D(h_{j}^{t})],$$

Consequently, the conditional discriminator optimized by the entropy-based loss function enables the entropy minimization principle [28] during the training stage and encourages the production of predictions with high certainty, so the CDAN model can perform well on unlabeled test data.

#### 2.2.3. Optimization of CDAN

During the training stage, the parameters $\theta_{c}$ in the classifier aim to minimize the label prediction cross-entropy loss $L_{c}\left(\theta_{f},\theta_{c}\right)$, while the parameters $\theta_{d}$ in the conditional discriminator try to minimize the domain classification loss $L_{d}(\theta_{f},\theta_{d})$ at the same time. Furthermore, the parameters $\theta_{f}$ in feature extractors are optimized by minimizing the classifier loss $L_{c}\left(\theta_{f},\theta_{c}\right)$ to get discriminative features and by maximizing the discriminator loss $L_{d}(\theta_{f},\theta_{d})$ in order to obtain domain-invariant features and make these two feature distributions as similar as possible simultaneously. Therefore, it shows the adversarial learning between the classifier and the conditional discriminator to generate common motor-imagery EEG features and domain-invariant representations. On the basis of the above analysis, the overall loss function of CDAN is expressed as:

(8) $$L(\theta_{f},\theta_{c},\theta_{d})=L_{c}\left(\theta_{f},\theta_{c}\right)-\lambda L_{d}(\theta_{f},\theta_{d}),$$

where *λ* is a hyper-parameter that controls the trade-off between the two objectives during training. 

As is shown in Figure 1, we insert the GRL before the conditional discriminator. During the forward propagation stage, the GRL makes no actions to the gradient, so the loss is calculated as common. However, in the backpropagation step, the GRL reverses the gradients in the discriminator by multiplying with a negative scalar, which is −λ, here. Then the reversed gradients will be passed to the preceding layer. As is proven in [35], the implementation of the GRL is very simple because of existing popular deep learning packages like Pytorch, as it just needs to multiply by a constant in the backprop step, and the parameter updating is trivial.

Generally, the optimal parameters ${\hat{\theta }}_{f},{\hat{\theta }}_{c},{\hat{\theta }}_{d}$ are learned by minimizing and maximizing the loss function of $L(\theta_{f},\theta_{c},\theta_{d})$ in Equation (8) iteratively. Firstly, we update the parameters of $\theta_{f}$ and $\theta_{c}$ by minimizing the loss function as follows:

(9) $$({\hat{\theta }}_{f},{\hat{\theta }}_{c})=\arg\;\underset{\theta_{f},\theta_{c}}{\min}L(\theta_{f},\theta_{c},{\hat{\theta }}_{d}).$$

Then, after obtaining the optimal parameters of ${\hat{\theta }}_{f}$ and ${\hat{\theta }}_{c}$, the optimal parameters of ${\hat{\theta }}_{d}$ can be updated by maximizing the following function:

(10) $$({\hat{\theta }}_{d})=\arg\;\underset{\theta_{d}}{\max}L({\hat{\theta }}_{f},{\hat{\theta }}_{c},\theta_{d}).$$

As a result, the feature extractor will generate the feature representations, which can minimize the loss of classifier and maximize the loss of conditional discriminator simultaneously. When the trained optimal discriminator cannot distinguish the features from the source domain or target domain, we obtain the common motor-imagery EEG features that exist in both the source and target domain.

As for the search algorithm of optimal model parameters, we use mini-batch stochastic gradient descent with 0.9 momentum and set the learning rate updating according to the procedure of training by the following formula [28]:

(11) $$\alpha =\alpha_{0}{\left(1+u\cdot \tau \right)}^{-\beta },$$

where τ is the training progress linearly changing from 0 to 1. Here, we set $\alpha_{0}$ = 0.01, u = 10, and β = 0.75 as the operation in [28]. It was demonstrated that this schedule was optimized to promote convergence and a low error on the source domain. We summarize the optimization steps of CDAN in Algorithm 1 and the details about calculating gradients can be found in Appendix A.
**Algorithm 1:** Steps of training CDAN model for EEG decoding. **Input:** EEG signals and label $D_{s}={\left\{\left(X_{i}^{s},y_{i}^{s}\right)\right\}}_{i=1}^{n_{s}}$ from source subject, EEG signals $D_{t}={\left\{\left(X_{j}^{t}\right)\right\}}_{j=1}^{n_{t}}$ from target subject, source domain label set $D_{s}=\left\{1\right\}$, target domain label set $D_{t}=\left\{0\right\}$; 1. Initialize model parameters and *λ*; 2. **Repeat** 3. Calculate the classifier loss $L_{c}\left(\theta_{f},\theta_{c}\right)$; 4. Optimize the parameters of the classifier by:  $\theta_{c}\leftarrow \theta_{c}-\alpha \frac{\partial L_{c}}{\partial \theta_{c}},\theta_{f}\leftarrow \theta_{f}-\alpha \frac{\partial L_{c}}{\partial \theta_{f}}$ 5. Calculate the discriminator loss $L_{d}(\theta_{f},\theta_{d})$; 6. Optimize the parameters of the domain discriminator by:  $\theta_{d}\leftarrow \theta_{d}-\alpha \frac{\partial L_{d}}{\partial \theta_{d}},\theta_{f}\leftarrow \theta_{f}+\alpha \lambda \frac{\partial L_{d}}{\partial \theta_{f}}$; 7. Adjust learning rate α as Equation (11); 8. **Until** the iterations satisfy the predefined condition; **Output:** 9. Learned parameters: $\theta_{d},\theta_{c},\theta_{f}$.

## 3. Experiments and Results

### 3.1. EEG Data

High gamma dataset (HGD) is a four-class motor-imagery EEG dataset which was collected from 14 subjects (8 males and 6 females) aged from 23 to 31. When recording each trial, an arrow cue was displayed on the screen to instruct the subject to perform either a left hand, right hand, foot, or rest motor imagery task. The EEG signals were recorded by 128 electrodes and the sampling rate was 500 Hz. For each subject, there were about 880 trials in the training set and approximate 160 trials in the test set respectively. As in the strategy in [24], only time-series obtained from 44 EEG electrodes which have high correlation to the motor imagery movements tasks were employed. We selected each trail by using the same time window [−0.5 s, 4 s] (relative to the cue onset) on the whole time series and resample the signals to 250 Hz, as in the preprocessing procedure in [12]. Therefore, the same deep learning architectures, such as deep ConvNet and Residual ConvNet in that research, can be used directly for comparison in later experiment sections. Furthermore, the length of the input trials was reduced to improve computing efficiency. Finally, we obtained each sample with 44 channels and 1125 time points. More details about this dataset can be seen in the supplementary material in [12].

### 3.2. Overall Comparison

In this section, we will implement several experiments on the HGD dataset. In order to evaluate the performance of the proposed CDAN method, we adopt evaluation metrics including accuracy, precision, recall, and the F1-score. Our proposed CDAN is compared with the algorithms as follows:

1. FBCSP is chosen as the baseline. This is a commonly used method to classify motor imagery EEG signals and has won several movement-related EEG decoding competitions [17].
2. Shallow ConvNet is proposed in [12], which contains two convolutional layers, a squaring nonlinearity layer, a mean pooling layer, and a logarithmic activation function.
3. Deep ConvNet is proposed in [12] as well. It is a deep convolutional neural network that contains four regular layers, and the deep ConvNet gains the state-of-the-art performance on the HGD dataset.
4. Residual ConvNet is a much deeper ConvNet compared with deep and shallow ConvNets. It is implemented by residual connection, but it produces a slightly poorer performance than the deep and shallow ConvNets.
5. Hybrid ConvNet concatenates output feature maps of Shallow ConvNet and Deep ConvNet into one layer and then the feature maps are fed into a new fully connected layer for classification.
6. CDAN-1 is a simplified version model compared with CDAN. It is implemented by using Equation (4) instead of Equation (7) as the loss function of conditional domain discriminator. This means that the entropy-based weights are removed, and each EEG trial takes the same importance during training.
7. The DAN model is a common domain adaptation network without conditioning. It is constructed by taking feature f instead of conditional feature f⊗g as the input of the domain discriminator.

From Table 1, we can see that the proposed CDAN method achieves the best recognition result among these various methods. The average accuracy of the baseline method FBCSP and Deep ConvNet reaches 91.2% and 92.5%, respectively. In particular, the CDAN achieves the highest accuracy of 95.3% for its ability to extract robust domain-invariant EEG representations. In addition, from Table 1, it is observed that the simplified domain adaptation models like DAN and CDAN-1 achieve more encouraging results (93.6% and 94.3%) than the baseline method. Furthermore, in terms of other evaluation metrics, such as the precision, recall, and F1-score, the CDAN model obtains the value of 95.2%, 95.3%, and 0.952 respectively. The CDAN model’s performance is more competitive than other compared methods. The results in the experiments prove the outstanding capability of the CDAN model to classify motor imagery EEG signals.

### 3.3. Efficiency of Conditional Domain Adaptation

**Figure 3:**
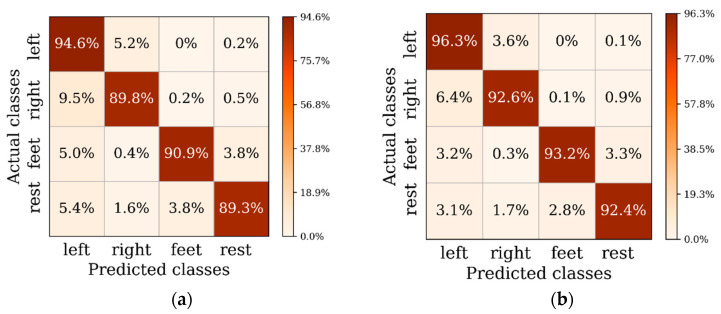

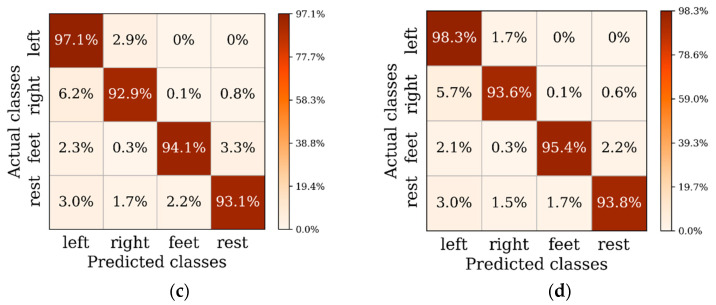
Confusion matrixes obtained by: (**a**) FBCSP; (**b**) Shallow ConvNet; (**c**) Deep ConvNet; (**d**) CDAN.

In this section, we compare the result of DAN, CDAN-1, and CDAN in detail to demonstrate the efficiency of the proposed entropy-based condition domain adaptation method. From Table 2, we can see that the three proposed methods obtain 93.6%, 94.3%, and 95.3% respectively. Note, that for the test set of the 14th subject, the signals from about half of the electrodes lost meaning, so all these methods gained slight lower accuracies on the 14th subject. However, our methods that reach the decoding accuracy of no lower than 85% is still competitive. For almost all the subjects, the CDAN gained better decoding results than DAN, which means that the conditional domain adaptation method improves the decoding performance efficiently, as multilinear conditioning can capture the cross-covariance between features and classes, which are crucial to domain adaptation. On the other hand, the CDAN outperforms the CDAN-1 and get 0.7% higher accuracy, which elucidates that the entropy-based weights are useful for the training of CDAN. It can be explained that the entropy weight is approximately 1 if the prediction is correct, and on the other hand, it would be approximately 0 if the prediction is incorrect or uncertain. Hence, the entropy conditioning technique can give priority to easy-to-transfer examples and encourage the output of certain predictions. This reveals the power of the entropy conditioning to guarantee example transferability. In addition, we try to exploit the efficacy of the entropy conditional domain adaptation method for a specific class of tasks using confusion matrixes on the test set. Figure 3 shows the confusion matrixes of FBCSP, DAN, CDAN-1, and CDAN. In these confusion matrixes, the values located in the diagonal line are the percentage of correctly predicted samples on each motor imagery classification task. It can be seen can see that our proposed CDAN method which take multilinear and entropy conditioning features gains better decoding performance on the four-class motor imagery tasks.

### 3.4. Influence of Hyperparameters

**Figure 4:**
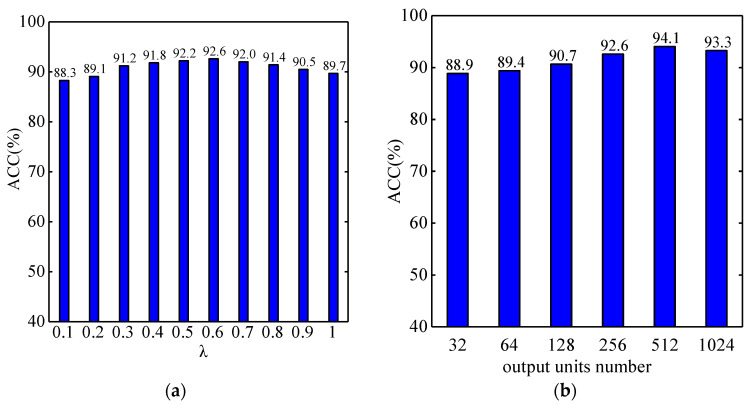

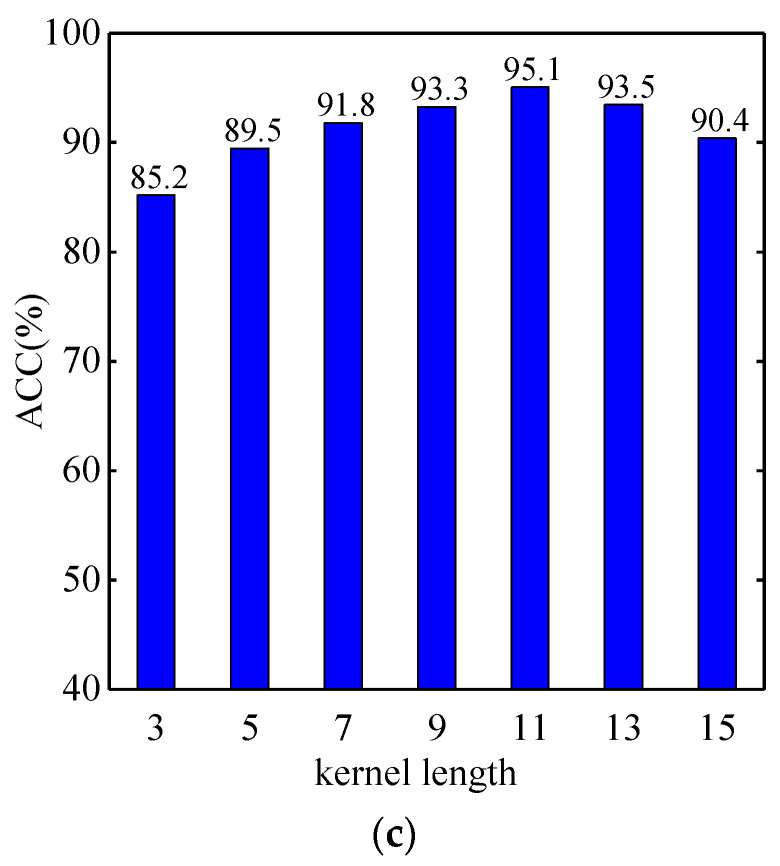
The accuracy obtained by CDAN with different hyperparameters including (**a**) *λ*; (**b**) number of output units and (**c**) kernel length.

In this section, we implement experiments to analyze the impact of different parameters in the CDAN. The cross validation is implemented to find key hyperparameters, such as the tradeoff parameters *λ*, the number of output units in feature extractor, and the kernel length in Dense ConvNet. First, we observe the impact of tradeoff parameters *λ* with 256 output units of feature extractor. Figure 4a shows the classification accuracy when setting *λ* from 0 to 1. It can been seen that when *λ* is 0.6, the CDAN gains the best result with 92.6% on the validation set. Then, we select the optimal number of output units with *λ* fixed to 0.6. Figure 4b shows the variation of the classification accuracy when we set the output number as 32, 64, 128, 256, 512, and 1024, respectively. The CDAN achieves an optimal performance with 94.1% accuracy on the validation set when we set the output units at 512. Therefore, in later experiments, we observe the impact of the convolutional kernel length with output units fixed at 512 and *λ* as 0.6. Figure 4c illustrates that the CDAN obtains better results with the kernel length at 11.

From these three figures, it can be seen that the decoding accuracy of the CDAN first goes up and then down as these parameters vary. Besides, we can also observe that the CDAN can perform stably under a wide range of parameters and archive better EEG decoding accuracy than the baseline method FBCSP.

### 3.5. Feature Correlation Maps

**Figure 5:**
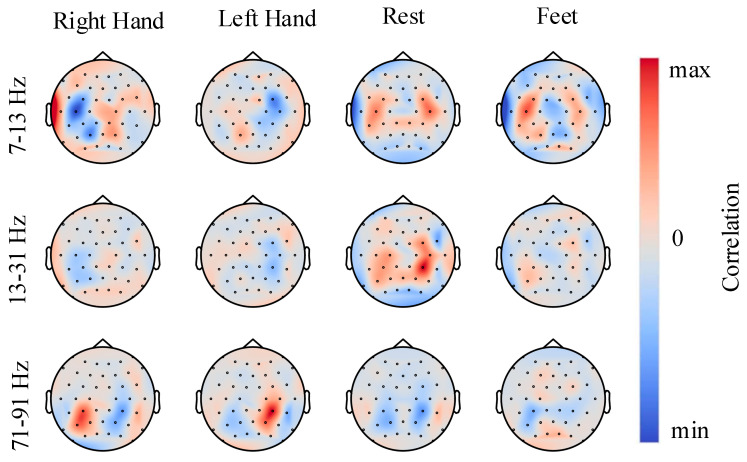
Feature correlation maps.

In this section, we apply the visualization method in [12] to investigate the correlation between the input signals and network predictions for the α (7–13 Hz), β (13–31 Hz), and γ (71–91 Hz) frequency bands. Firstly, Fourier transformation (FFT) is used on all EEG trials and transforms them to the frequency domain. Then, Gaussian noise (with a mean of 0 and variance 1) is randomly added to the amplitudes. Next, we retransformed the signals to time domain by inverse FFT and computed the predictions of the CDAN model for these EEG trials before and after the perturbation. Finally, the changes in the input amplitudes of each electrode, which is the perturbation we added, were correlated with the changes in the prediction results of the CDAN model. The results are visualized in Figure 5 by scalp maps and the colors in the maps represent the correlation of input amplitude and the corresponding changes of the model output. Plausible results can be seen from Figure 5, for example, in the α and β frequency bands, the left (right) sensorimotor hand areas show negative correlations with the right (left) hemisphere. This means that the amplitudes increase in the left (right) areas leads to a prediction decrease for the right (left) hand class. Then, on the γ frequency band, there is an opposite trend compared with the α and β bands. Furthermore, for the rest class, positive correlations are mainly shown in the α and β bands, while negative correlations are described in the gamma band. In total, these scalp maps show spatial distributions which are expected for different motor imagery tasks in the α, β, and γ bands and reflect the reliability of the CDAN model for EEG classification. More explanations and details about this visualization technique can be found in [12].

## 4. Conclusions

In this paper, a novel CDAN method for motor imagery EEG decoding is proposed. Firstly, the feature extractor is employed to learn discriminative features automatically from raw EEG signals. Then, the extracted features are fed into the label classifier and domain discriminator, respectively. The proposed CDAN method conditions the adversarial domain adaptation on discriminative information to enable the alignment of different distributions across source and target domains in the dataset and assign different weights on each EEG trail. By training the label classifier and domain discriminator iteratively, the feature extractor learns to capture domain-invariant features for classification. The results in the experiment show that the proposed CDAN could obtain a better decoding performance compared to other state-of-the-art methods. Additionally, the proposed CDAN model is based on the domain adaptation technique, so it needs less data to learn optimal parameters in the model during the training steps. Therefore, it costs less time to collect training data for one subject and is more convenient to deploy in online BCI systems. Although it needs hours to train the CDAN model, the testing time is acceptable, as described in Appendix B.

The limitation is that our method is only implemented on the visual motor imagery dataset, while it is observed that kinesthetic motor imagery may gain more competitive performance when applied to BCI systems. In the future, we will try to use the CDAN model on kinesthetic motor imagery EEG datasets and compare the difference between them. In addition, we will focus on the research about learning more effective features from EEG data by using other advanced domain adaptation techniques, such as the maximum mean discrepancy.

## Figures and Tables

**Table 1:** Overall comparison.

Method	Acc (%)	Precision (%)	Recall (%)	F1-Score
FBCSP	91.2	91.6	91.2	0.914
Shallow ConvNet	89.3	89.5	89.3	0.894
Deep ConvNet	92.5	92.7	92.4	0.926
Hybrid ConvNet *	91.9	92.1	91.8	0.920
Residual ConvNet *	88.8	88.9	88.8	0.888
DAN	93.6	93.8	93.5	0.936
CDAN-1	94.3	94.4	94.3	0.943
CDAN	95.3	95.2	95.3	0.952

* denotes the experiment results are obtained by our own reimplementation.

**Table 2:** Results of all subjects obtained by our methods.

Subject	DAN	CDAN-1	CDAN
1	92.4	93.7	95.0
2	94.2	95.3	96.3
3	94.5	94.9	96.3
4	97.0	97.4	98.1
5	98.6	98.6	99.4
6	93.5	94.5	95.0
7	92.3	93.2	93.7
8	97.7	98.4	98.8
9	95.8	96.8	97.5
10	90.3	91.2	92.5
11	90.9	91.3	92.5
12	94.1	94.7	95.6
13	93.4	94.3	95.6
14	85.3	86.2	87.5
Average	93.6	94.3	95.3

## Appendix A

In a neural network model, the outputs of the former layer are connected as the inputs of the next layer. The gradients of each layer are calculated by the chain rule. For example, when optimizing the discriminator, the partial derivative of the loss function to the middle layer outputs could be calculated as:

(A1) $$\frac{\partial L_{d}}{\partial a_{i}^{l}}=\sum_{j}\frac{\partial L_{d}}{\partial a_{j}^{l+1}}\frac{\partial a_{j}^{l+1}}{\partial a_{i}^{l}},$$

where $a_{i}^{l}$ is the $i^{th}$ output of the layer *l*, and *j* is number of elements in the layer *l* + 1. The CDAN model contain two types of layers: the convolutional and fully connected layers, which we will discuss below.

First the gradients are calculated on the fully connected layer. The output of the $l^{th}$ layer is denoted as $a_{i}^{l}=f(\omega_{i}a_{i}^{l-1}+b)$, so the partial derivative can be expressed as:

(A2) $$a_{i}^{l}=\omega_{i}f^{\prime }(\omega_{i}a_{i}^{l-1}+b),$$

where $\omega_{i}$ represents the weight in this layer and $f^{\prime }(\omega_{i}a_{i}^{l-1}+b)$ is the derivative of the activation function.

Then, as for convolutional layers, only a 1-D convolutional kernel is employed in the proposed CDAN model. Assume that the output of the layer *l* is a matrix, which is connected to next layer by a convolutional kernel with size of *k* × 1. The gradients are obtained by partial derivative as:

(A3) $$\begin{array}{ll} \frac{\partial L_{d}}{\partial a_{i}^{l}} & =\sum_{n=0}^{k-1}\frac{\partial L_{d}}{\partial a_{i-n}^{l+1}}\frac{\partial a_{i-n}^{l+1}}{\partial a_{i}^{l}}\\ & =\sum_{n=0}^{k-1}\frac{\partial L_{d}}{\partial a_{i-n}^{l+1}}\omega_{n}^{l+1}f^{\prime }(a_{i}^{l}) \end{array}$$

where $\omega_{n}^{l+1}$ denotes the $n^{th}$ element of the convolutional kernel, and $f^{\prime }(a_{i}^{l})$ is the derivative of activation function.

## Appendix B

We performed the CDAN model on NVIDIA Tesla V100 GPU with 11 GB memory. Our computing machine has Intel(R) Xeon(R) Gold 5118 CPU @ 2.30 GHz with 12 cores and 256 GB RAM. The CDAN model were implemented by the popular Pytorch framework, and the preprocessing of EEG data were implemented by MNE library. The training time of the CDAN model for each subject is listed in Table A1. Though the training of the CDAN model is time-consuming, its prediction speed is still competitive (about 0.3 s) when testing on a single trial of EEG data.

**Table A1:** Training time of the CDAN model for each subject.

**Subject**	1	2	3	4	5	6	7	8
**Time (hour: min)**	1:28	1:28	1:59	2:18	1:58	1:41	1:39	1:36
**Subject**	9	10	11	12	13	14	average	
**Time (hour: min)**	1:42	1:46	2:34	2:14	2:18	2:18	1:53	
